# Complete Genome Sequence of the Attenuated Novobiocin-Resistant *Streptococcus iniae* Vaccine Strain ISNO

**DOI:** 10.1128/genomeA.00510-14

**Published:** 2014-05-29

**Authors:** Julia W. Pridgeon, Dunhua Zhang, Lee Zhang

**Affiliations:** a Aquatic Animal Health Research Unit, USDA, Auburn, Alabama, USA; b Genomics and Sequencing Laboratory, Department of Entomology and Plant Pathology, Auburn University, Auburn, Alabama, USA

## Abstract

*Streptococcus iniae* ISNO is an attenuated novobiocin-resistant vaccine strain. Its full genome is 2,070,182 bp in length. The availability of this genome will allow comparative genomics to identify potential virulence genes important for pathogenesis of *S. iniae* and potential mechanisms associated with novobiocin resistance in this strain.

## GENOME ANNOUNCEMENT

The Gram-positive bacterium *Streptococcus iniae* is a zoonotic pathogen that causes infections in both humans and fish (1–3). As a serious marine and freshwater fish pathogen, *S. iniae* causes significant economic losses to aquaculture (4, 5). This pathogen was originally isolated from Amazon freshwater dolphin (*Inia geoffrensis*) in 1976 (6). However, *S. iniae* causes diseases not only to dolphin, but also to many fish species, including rainbow trout (*Oncorhynchus mykiss* [7]), barramundi (*Lates calcarifer* [8]), red drum (*Sciaenops ocellatus* [9]), flounder (*Paralichthys* spp. [10, 11]), and tilapia (*Oreochromis* spp. (12)]. A highly virulent strain, *S. iniae* ISET0901, was cultured from diseased Nile tilapia (*Oreochromis niloticus*) during a disease outbreak in 2005 (13). Through selection for resistance of *S. iniae* ISET0901 to novobiocin, an attenuated vaccine strain, *S. iniae* ISNO, was obtained (13). The vaccine strain *S. iniae* ISNO was found to be totally safe to Nile tilapia at various exposure doses and in back passage safety studies (13). In addition, the vaccine strain *S. iniae* ISNO offered Nile tilapia significant protection against *S. iniae* infections at a wide range of efficacious immunization doses (13). Furthermore, the vaccine strain *S. iniae* ISNO provided tilapia significant protection up to 6 months after a single vaccination (13). Compared to the virulent parent *S. iniae* ISET0901, the vaccine strain *S. iniae* ISNO was ~1,000-fold resistant to novobiocin. However, what changes occurred at the genomic DNA level were unknown. Therefore, the complete genome sequence of *S. iniae* ISNO was determined in this study.

The genome of *S. iniae* ISNO was sequenced using the Illumina 1500 HiSeq platform. BioNumerics (Applied Maths) was used to assemble a total of 15,690,266 sequence reads with an average length of 92.32 bp (estimated 700× coverage) using the complete genome of *S. iniae* SF1 (GenBank accession no. CP005941) as reference. The assembled genome of *S. iniae* ISNO is 2,070,182 bp, with G+C content of 36.8%. RNAmmer (14) predicted 12 copies of ribosomal RNA (4 copies of 5S RNA, 16S RNA, and 23S RNA, respectively), similar to reference genome *S. iniae* SF1 (15). The RAST server (16) predicted 1,980 coding sequences belonging to 303 subsystems, including 291 involved in carbohydrate catabolism, 149 in protein metabolism, 135 in synthesis of amino acids and derivatives, 114 in cell wall and capsule synthesis, 95 in RNA metabolism, and 92 in DNA metabolism (including 77 in cofactors, vitamins, prosthetic groups, or pigments; 63 in nucleoside and nucleotide synthesis; 62 in fatty acid and lipid synthesis; 52 in virulence, disease, and defense; 47 in membrane transport; 37 in stress response; 31 in phosphorus metabolism; 26 in regulation and cell signaling; 7 in secondary metabolism; and 2 in motility and chemotaxis).

### Nucleotide sequence accession number.

The complete genome sequence of *S. iniae* ISNO was deposited at GenBank under the accession no. CP007587.
